# Supplementary material for: Sequence – dynamics – function relationships in protein tyrosine phosphatases
Source: QRB Discov. 2024 Jan 24;5:e4. doi: 10.1017/qrd.2024.3 (PMC11058592; doi:10.1017/qrd.2024.3)
Supplement: Crean et al. supplementary material [file S2633289224000036sup001.pdf]

Supporting Information for:

Sequence–Dynamics–Function Relationships in Protein Tyrosine Phosphatases

Rory M. Crean,[1] Marina Corbella,[1,2] Ana R. Calixto,[1,3] Alvan C. Hengge[4,*] and S. C. L. Kamerlin[1,5*]

1. Department of Chemistry – BMC, Uppsala University, BMC Box 576, S-751 23 Uppsala, Sweden.
2. Departament de Química Inorgànica i Orgànica (Secció de Química Orgànica) & Institut de Química Teòrica i Computacional (IQTCUB), Universitat de Barcelona, Martí i Franquès 1, 08028 Barcelona, Spain.
3. LAQV, REQUIMTE, Departamento de Química e Bioquímica, Faculdade de Ciências, Universidade do Porto, Rua do Campo Alegre, s/n, 4169-007 Porto, Portugal.
4. Department of Chemistry and Biochemistry, Utah State University, Logan, Utah 84322-0300, USA.
5. School of Chemistry and Biochemistry, Georgia Institute of Technology, 901 Atlantic Drive NW, Atlanta, GA 30332, USA.

Corresponding author email addresses: alvan.hengge@usu.edu, skamerlin3@gatech.edu

Table of Contents

S1. Supplementary Methods S3

System Preparation for Empirical Valence Bond Simulations S3

MD Equilibration Procedure S4

System Preparation for Targeted Molecular Dynamics Simulations S5

Molecular Dynamics Simulations and Analysis S5

S2. Supplementary Figures S8

S3. Supplementary Tables S23

S4. Supplementary References S29

S1. Supplementary Methods

System Preparation for Empirical Valence Bond Simulations

Since the WPD-loop containing the catalytic aspartic side-chain is in its catalytically-inactive open conformation in the crystal structures available for both chimeras (PDB IDs: 6DR7 (Moise et al. 2018) and 6DT6 (Moise et al. 2018), respectively), we used wild-type YopH crystal structure (PDB ID: 2I42 (Berman et al. 2000)) and modelled the corresponding substitutions *in silico*. The rotamers for the residues that were lying in a position of the loop that is in the same conformation as in the Chimera 2 and 3 crystal structures (PDB IDs: 6DR7 (Moise et al. 2018) and 6DT6 (Moise et al. 2018), respectively) were chosen to match the conformation present in the crystal structure, as well as being coincident with the highest probability rotamer from Dunbrack 2010 rotamer Library (Shapovalov and Dunbrack Jr. 2011) as implemented in UCSF Chimera, v. 1.14 (Pettersen et al. 2004). Other side-chains were chosen taking the highest probability rotamer from Dunbrack 2010 rotamer Library (Shapovalov and Dunbrack Jr. 2011). The phosphoenzyme intermediate and nucleophilic water molecule were constructed/placed manually into the active site in order to optimize the position of the water molecule for nucleophilic attack on the phosphate. In addition, two crystallographic waters from the original crystal structures were removed to prevent steric clashes with new bulky sidechain from the mutated Phe357. A complete list of all ionized residues and histidine protonation patterns for each system can be found in the Supporting Information (**Table S4**), and full simulation protocols can be found in our prior work (Crean et al. 2021; Shen et al. 2022). All relevant input and parameter files necessary to reproduce our calculations, have been uploaded as a data package to Zenodo, DOI: 10.5281/zenodo.7892058.

MD Equilibration Procedure

To prepare our systems for production molecular dynamics (MD) simulations, we followed a standard protocol, which we have previously used to model wild-type YopH (Crean et al. 2021). This can be described as follows: (1) energy minimization of hydrogen atoms and solvent molecules (including counterions) with 500 steps of steepest descent and then 500 steps of conjugate gradient minimization. To do this, all other atoms were held in place using 10 kcal mol^{-1} Å^{-2} positional restraints. (2) Heating of the system from 50 to 300 K in the NVT ensemble over 200 ps (1 fs timestep, 1 ps^{-1} collision frequency) with the previously described positional restraints retained. (3) Energy minimization (again 500 steps of both steepest descent and then conjugate gradient minimization) with 5 kcal mol^{-1} Å^{-2} positional restraints on the C_α-atoms. (4) Heating from 25 K to 300 K over 500 ps in the NVT ensemble (1 fs timestep), with the C_α-atom restraints described in step 3 retained. (5) A series of 5 stepwise simulations in which the C_α-atom restraints were decreased from 5 kcal mol^{-1} Å^{-2} to 0 kcal mol^{-1} Å^{-2} in 1 kcal mol^{-1} Å^{-2} steps. These simulations were performed in the NPT ensemble (298 K, 1 atm) with each step lasting 10 ps and using a 2 fs time step. (6) A final NPT simulation without restraints was ran lasting 1 ns. All simulation steps used the SHAKE algorithm (Ryckaert et al. 1977) to restrain all bonds to hydrogen atoms. NVT and NPT simulations used Langevin temperature control (Schneider and Stoll 1978) with a collision frequency of 1 ps^{-1}. The NPT simulations also used a Berendsen barostat (Berendsen et al. 1984) with the pressure relaxation time set to 1 ps. After this, simulations were then considered ready for production simulations. Replicas were generated by being assigned different random velocity vectors for both heating steps.

System Preparation for Targeted Molecular Dynamics Simulations

Crystal structures of Chimeras 2 and 3 in the wide-open conformation (PDB IDs: 6DT6 (Moise et al. 2018) and 6DR7 (Moise et al. 2018), respectively) were used as the starting structures for the tMD simulations. As there are no crystal structures of wild-type YopH in the wide-open conformation, we extracted two structures of wild-type YopH in this state from our prior study (Crean et al. 2021) in which we used Hamiltonian replica exchange molecular dynamics (HREX-MD) simulations (Bussi 2014) to sample the conformations available to wild-type YopH. The two structures selected are in the wide-open conformation and one structure was selected to represent Chimera 2's wide open conformation, with the other selected to represent Chimera 3's wide open conformation. The wild-type YopH structure representing the Chimera 3 wide open crystal structure (PDB ID: 6DT6 (Moise et al. 2018)) conformation has a backbone WPD-loop RMSD of 1.03 Å and the structure representing the wide open Chimera 2 crystal structure (PDB ID: 6DR7 (Moise et al. 2018)) conformation has backbone WPD-loop RMSD of 0.95 Å. The starting structures used for both conventional and targeted MD simulations are summarized in **Tables S5** to **S7**.

Molecular Dynamics Simulations and Analysis

Molecular dynamics (MD) simulations of wild-type YopH and Chimeras 3 and 2 were performed from all three major WPD-loop conformational states: the closed, open, and wide-open conformations. For each enzyme and conformational state, 15 replicas of 500 ns long production MD simulations were performed in order to generate data for analysis. MD and tMD simulations were primarily analysed using CPPTRAJ (Roe and Cheatham 2013) in order to calculate the relevant RMSD and RMSF values. RMS fitting prior to the RMSD and RMSF calculations were performed using a selection of stable secondary structure residues (to ensure a good fit throughout

the simulations), which were: 191-208, 246-251, 254-259, 264-277, 281-284, 288-296, 306-308, 311-324, 327-337, 344-351, 362-386, 389-392, 400-402, 408-420 and 429-440. The statistical significance of the ΔRMSF values calculated was determined using a two-sided *t*-test, using the RMSF values determined from the 15 replicas performed per system in each conformation. To account for the usage of many *t*-tests at once (which if left unchecked would result in many false positives), the Benjamini-Hochberg correction (Benjamini and Hochberg 1995) was applied, with the false discovery rate set to 5%.

To determine how the non-covalent interaction network differed between the conformational states we used our program Key Interactions Finder (KIF) (Crean et al. 2023). KIF makes use of the program PyContact (Scheurer et al. 2018) to first determine all non-covalent interactions present in a simulation before determining those that are associated with the conformational change of interest. KIF was used to identify differences in the non-covalent interaction network for two different enzymes in the same conformational state (a binary classification problem). This effectively meant labelling simulation frames according to which enzyme the simulation frame came from. Mutated residues were considered equivalent in the KIF analysis. The Jensen-Shannon distance metric was used to score each interaction (available from the statistical analysis package of KIF), with scores ≥ 0.3 analysed.

Simulation frames were only included in the KIF analysis if they were determined to belong to one of the 3 states of interest (closed, open and wide open). Here, we used crystal structures of wild-type YopH (PDB IDs: 2I42 (Berman et al. 2000) and 1YPT (Stuckey et al. 1994)) as reference structures to define the closed and open states, respectively. Frames with a WPD-loop C_α-atom RMSD ≤1.5 Å from each reference state were defined as belonging to these conformational states. For the wide-open conformation, if residue 358 was in an α-helical conformation (as defined by

the DSSP algorithm available within CPPTRAJ (Roe and Cheatham 2013)), the frame was considered part of the wide-open conformation.

S2. Supplementary Figures

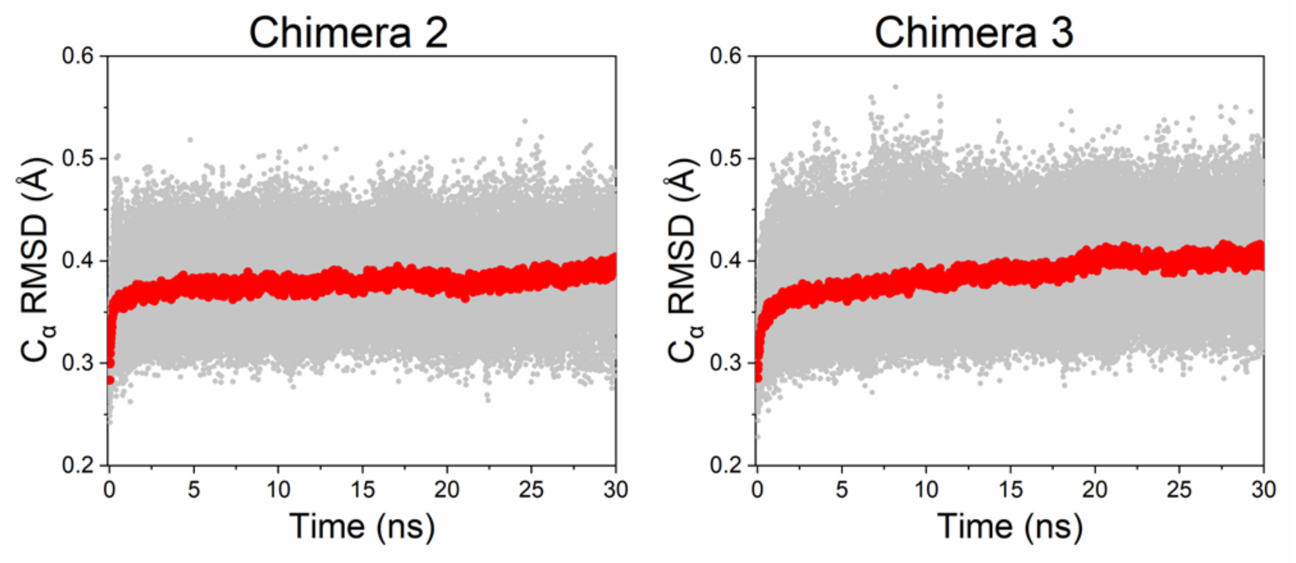

Figure S1. The root mean square deviations (RMSD, Å) of all C_α-atoms during our EVB equilibration MD simulations. Equilibration simulations were performed at the approximate EVB transition states ($\lambda = 0.5$) for both Chimeras in the second, rate-limiting, catalytic step. Data was collected every 10 ps from 30 replicas each of length 30 ns. The grey lines show the 30 individual runs, whilst the red line shows a rolling average RMSD from all 30 replicas. The low RMSD values observed are the consequence of using the surface constraint all atom solvent (SCAAS) model to describe our simulation sphere, as in prior work (for details, see refs. (Crean et al. 2021; Warshel and King 1985; Warshel and Weiss 1980)). This approach applies restraints to residues at the surface of the explicit simulation sphere and outside the explicit simulation sphere, restricting the mobility of regions further from the active site / reacting center. We also note that we have previously simulated WT-YopH (Crean et al. 2021), so these plots are not shown in the manuscript.

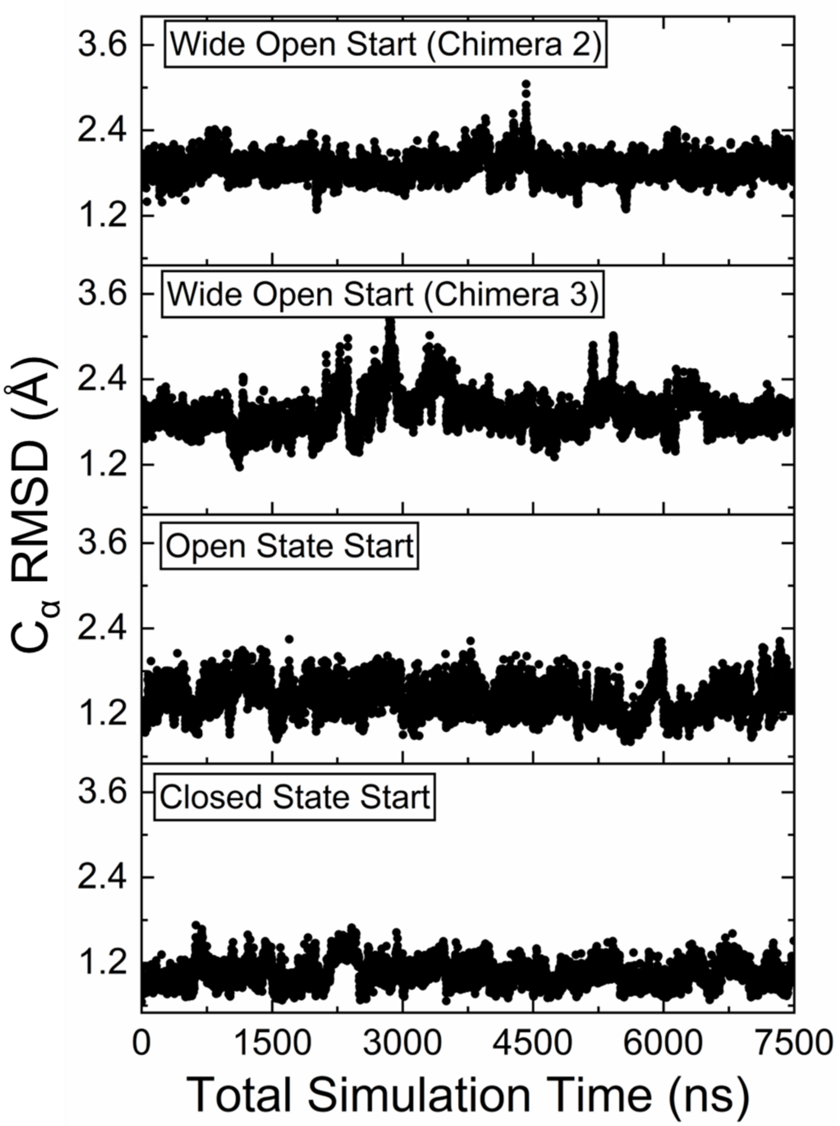

Figure S2. The root mean square deviations (RMSD, Å) of all C_α-atoms for our MD simulations of WT-YopH from four different starting structures. The starting structure used to create each panel (which refers to the WPD-loop conformation) is provided inside the panel. Each plot is made up of 15 replicas of 500 ns long MD simulations, to a total cumulative simulation time of 7.5 μs.

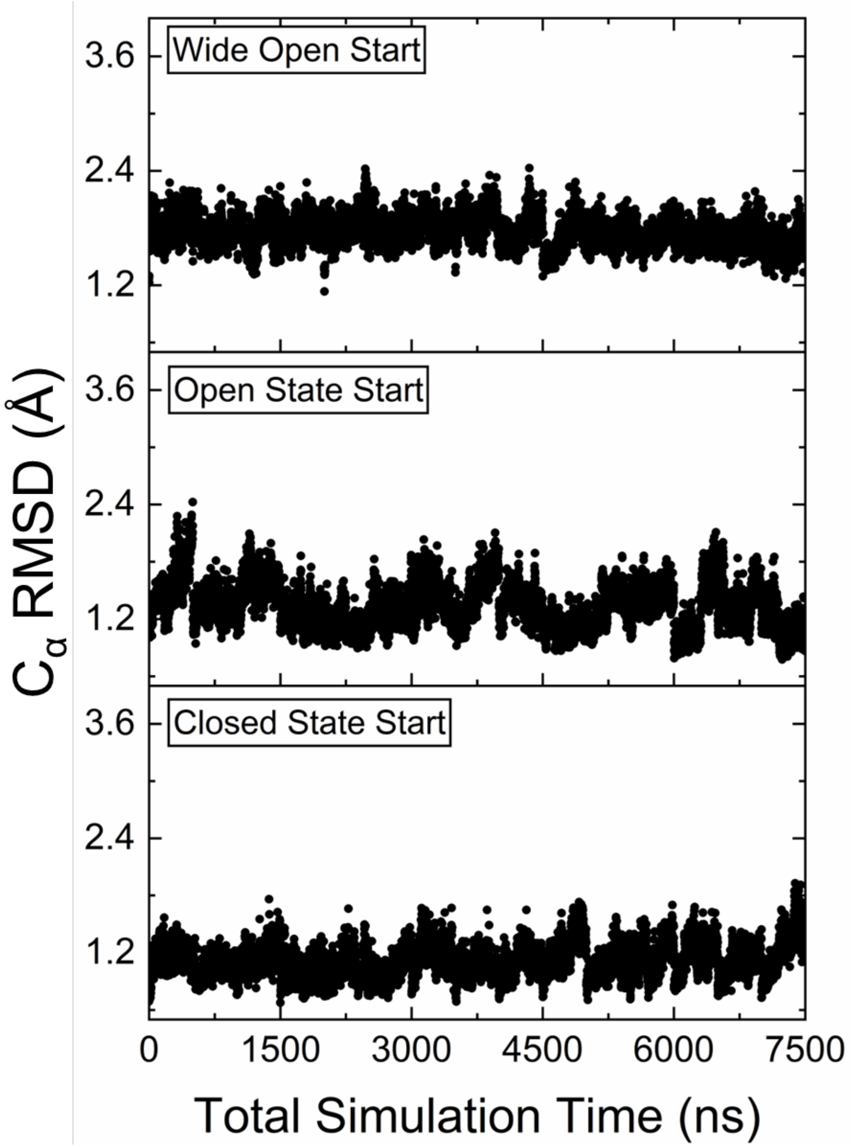

Figure S3. The root mean square deviations (RMSD, Å) of all C_α-atoms for our MD simulations of YopH Chimera 3 from three different starting structures. The starting structure used to create each panel (which refers to the WPD-loop conformation) is provided inside the panel. Each plot is made up of 15 replicas of 500 ns long MD simulations, to a cumulative simulation time of 7.5 μs.

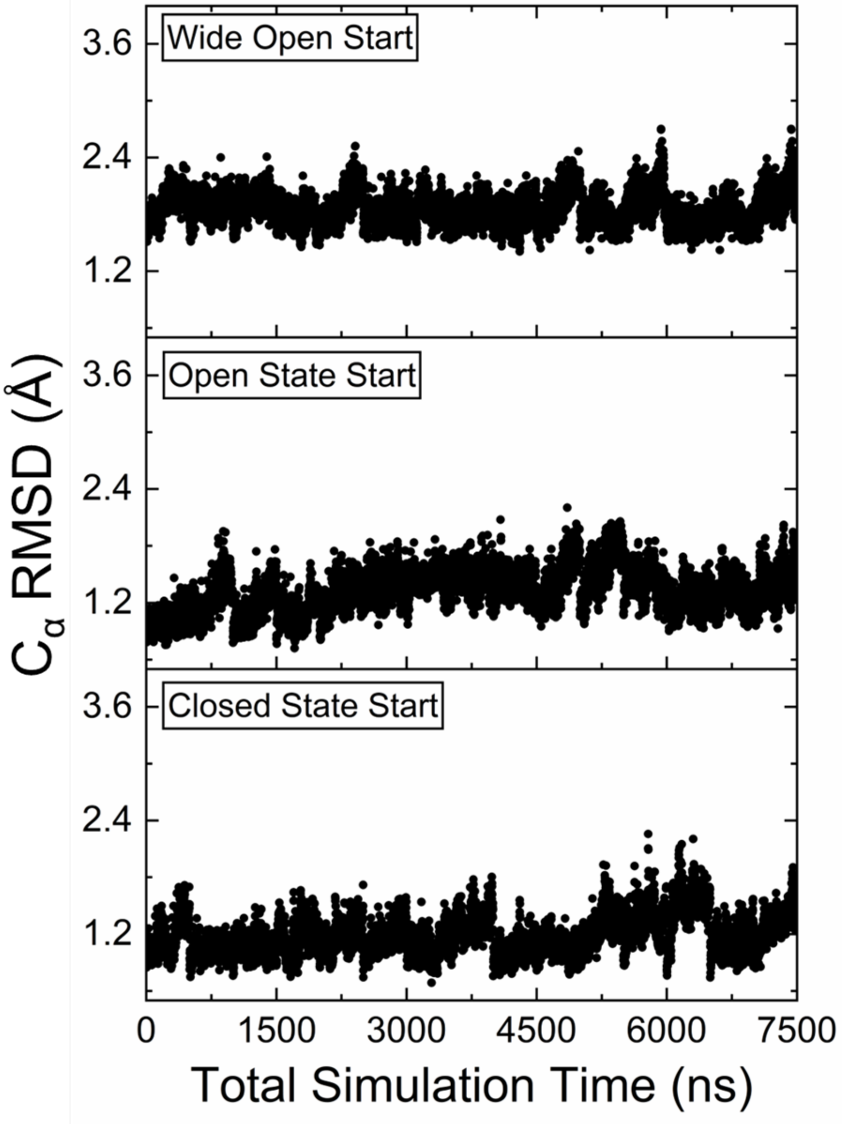

Figure S4. The root mean square deviations (RMSD, Å) of all C_{α}-atoms for our MD simulations of YopH Chimera 2 from three different starting structures. The starting structure used to create each panel (which refers to the WPD-loop conformation) is provided inside the panel. Each plot is made up of 15 replicas of 500 ns long MD simulations, to a cumulative simulation time of 7.5 μs.

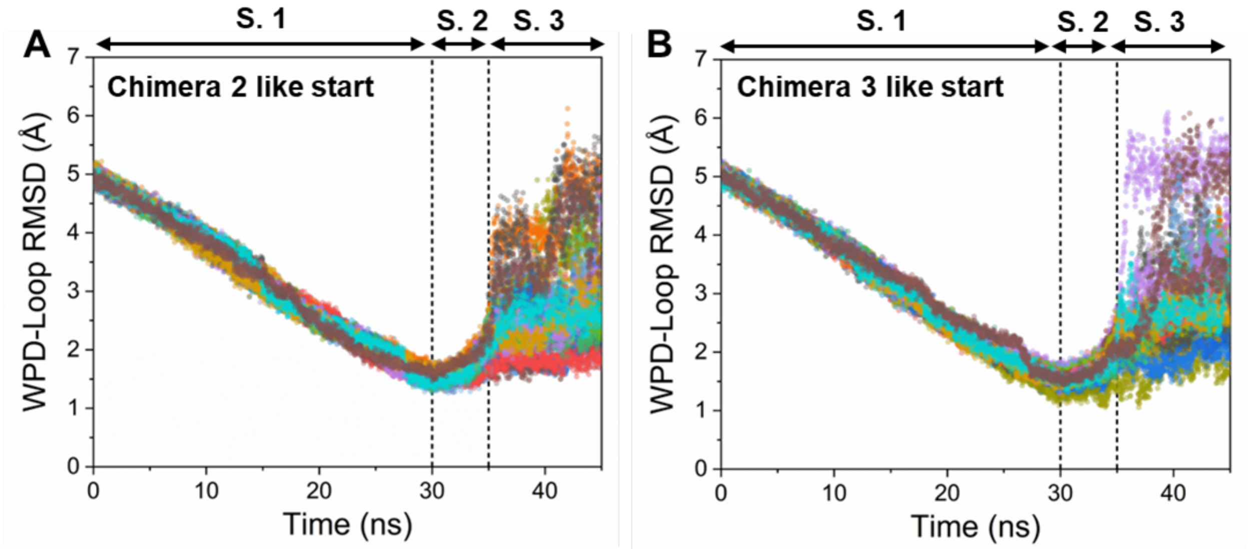

Figure S5. (**A**, **B**) The backbone RMSD to the closed WPD-loop conformation over the course of our targeted MD (tMD) simulations of wild-type (WT) YopH. Two "wide-open" WPD-loop conformations of WT-YopH were used and were obtained from a prior study (Crean et al. 2021) we performed in which we used HREX-MD (Bussi 2014) simulations to sample the conformational space available to WT-YopH. Panel **A** starts from a snapshot with a WPD-loop RMSD to the Chimera 2 wide-open structure of 0.95 Å (PDB ID: 6DR7 (Moise et al. 2018)), whilst panel **B** starts from a snapshot with a WPD-loop RMSD to the Chimera 3 wide-open structure of 1.03 Å (PDB ID: 6DT6 (Moise et al. 2018)). The markings above the graph indicate the three steps/phases involved in the tMD simulations: S. 1: Steering to the closed state with a constant force; S. 2: gradual removal of the steering force; S. 3: unrestrained MD simulations. See the **Methods** section for further details on the tMD simulation protocol used.

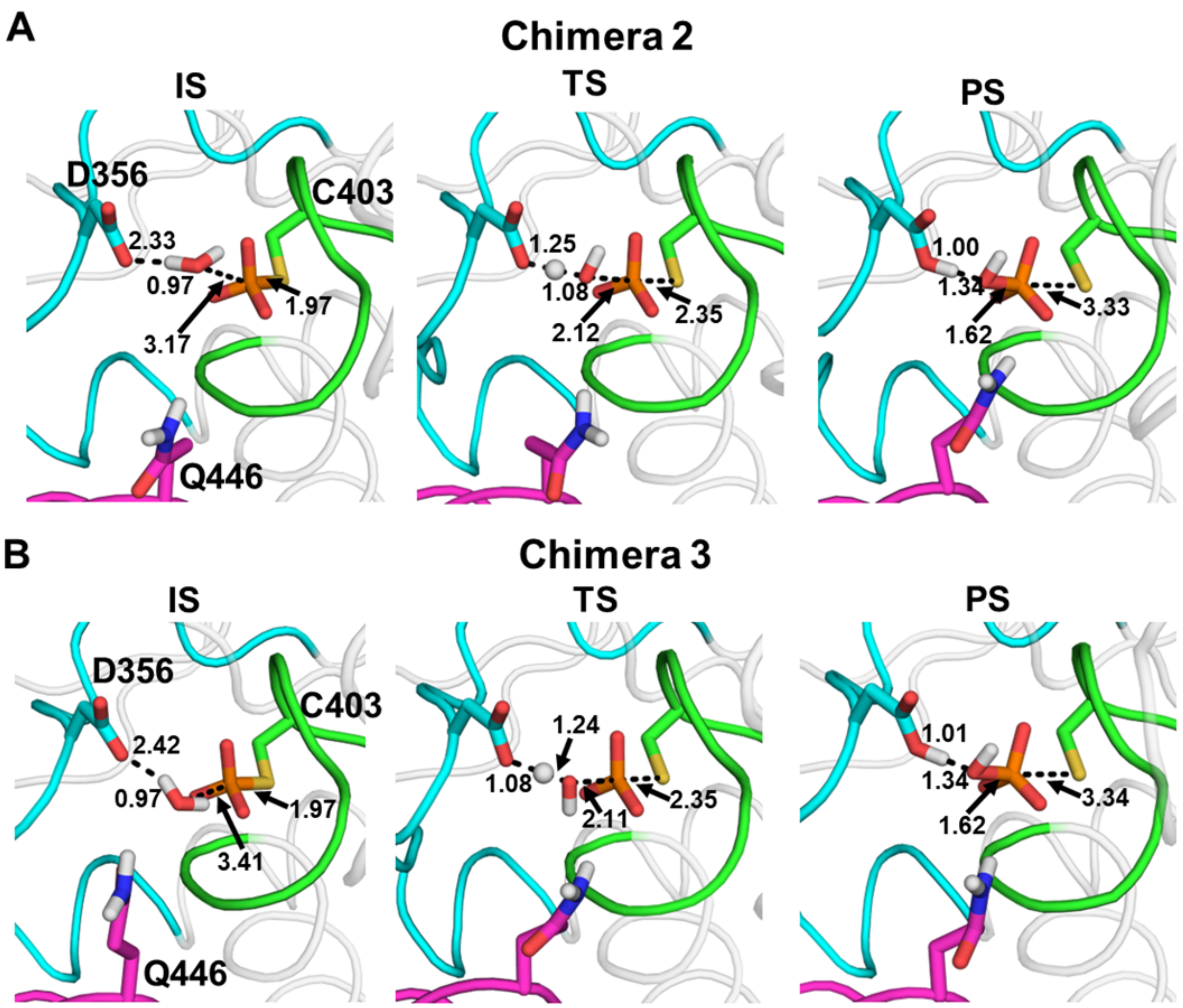

Figure S6. Representative structures from EVB simulations of the rate-limiting hydrolysis step of the reaction catalyzed by (**A**) Chimera 2 and (**B**) Chimera 3. The panels in **A** and **B** show, from left to right, the phospho-enzyme intermediate, the transition state for the hydrolysis step, and the final product complex, for the hydrolysis reaction for both enzymes. The structures shown here are the centroids of the top ranked cluster obtained from RMSD clustering of 30 individual EVB trajectories of each stationary or saddle point, performed as described in the **Supporting Information**. Average reacting distances for each catalytic step are also shown (see also **Table S2**).

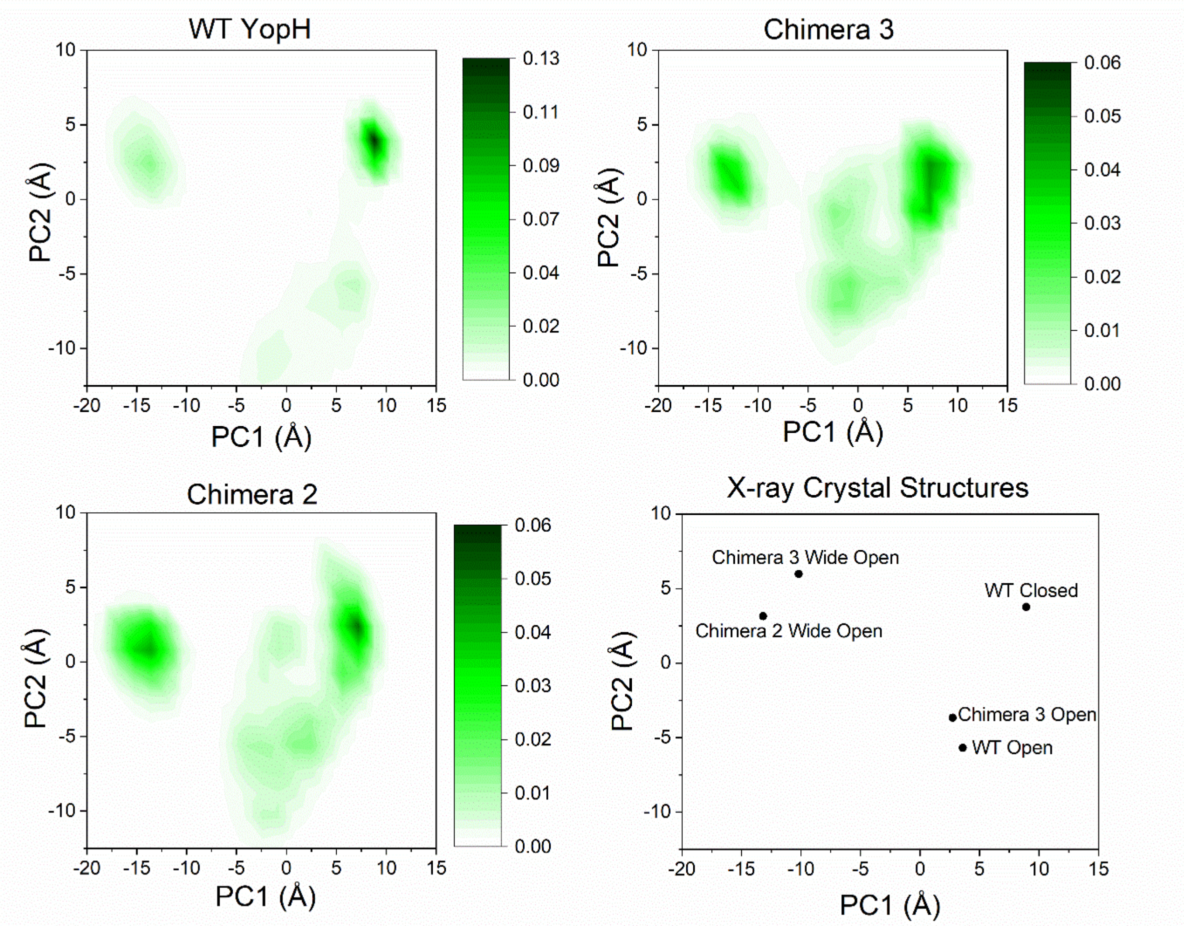

Figure S7. Projections of principal components (PC) 1 and 2 obtained from PC analysis (PCA) on the C_α-atoms of the WPD-loop for each enzyme. PCA was performed on all three systems combined (*i.e.,* PC1 and PC2 are the same for all systems) using 22.5 μs of data per system (500 ns long simulations of 3 different states with each state having 15 replicates). The histograms shown are normalized such that sum of all the bins is 1. The bottom right panel shows a projection of the coordinates of the 5 X-ray structures associated with this study (see **Tables S5-S7** for the crystal structures used) and their associated WPD-loop conformation (closed, open or wide open). The histograms show poor sampling between the defined states (especially for WT YopH), which is likely the reason why we were unable to build Markov State Models using this dataset.

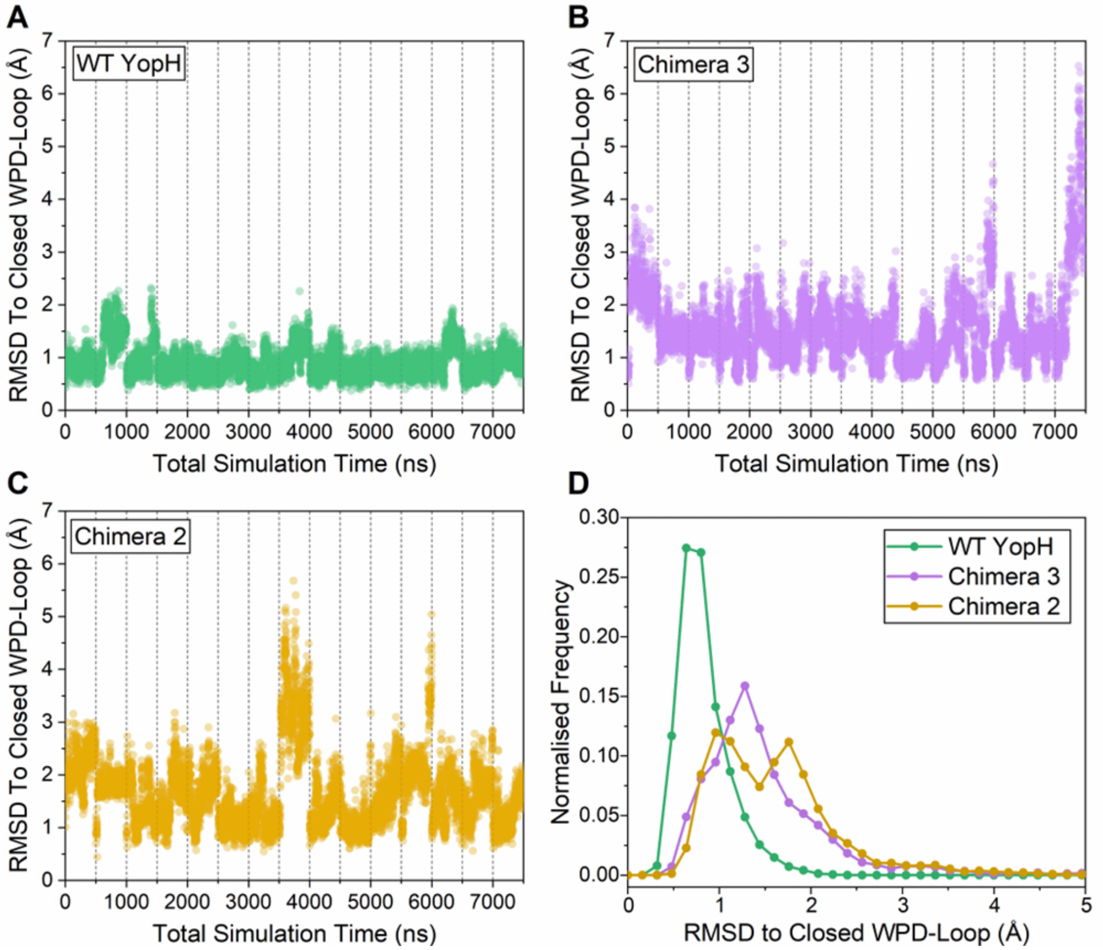

Figure S8. Evaluating the stability of the closed WPD-loop conformational state. (**A-C**) Traces of the WPD-loop C_α-atom RMSD (Å) to the closed crystal structure conformation for simulations of (**A**) wild-type (WT)YopH, (**B**) Chimera 3 and (**C**) Chimera 2 over the course of the simulations. Each panel depicts data from the 15 replicas of 500 ns long MD simulations performed, with the grey dotted lines representing the start of a new simulation replica. (**D**) Histograms of the WPD-loop Cα-atom RMSD to the closed WPD-loop conformational state, obtained from the data shown in panels **A-C**. Each histogram was constructed by sampling the conformation every 100 ps (75000 frames in total), and using a bin width of 0.16 Å.

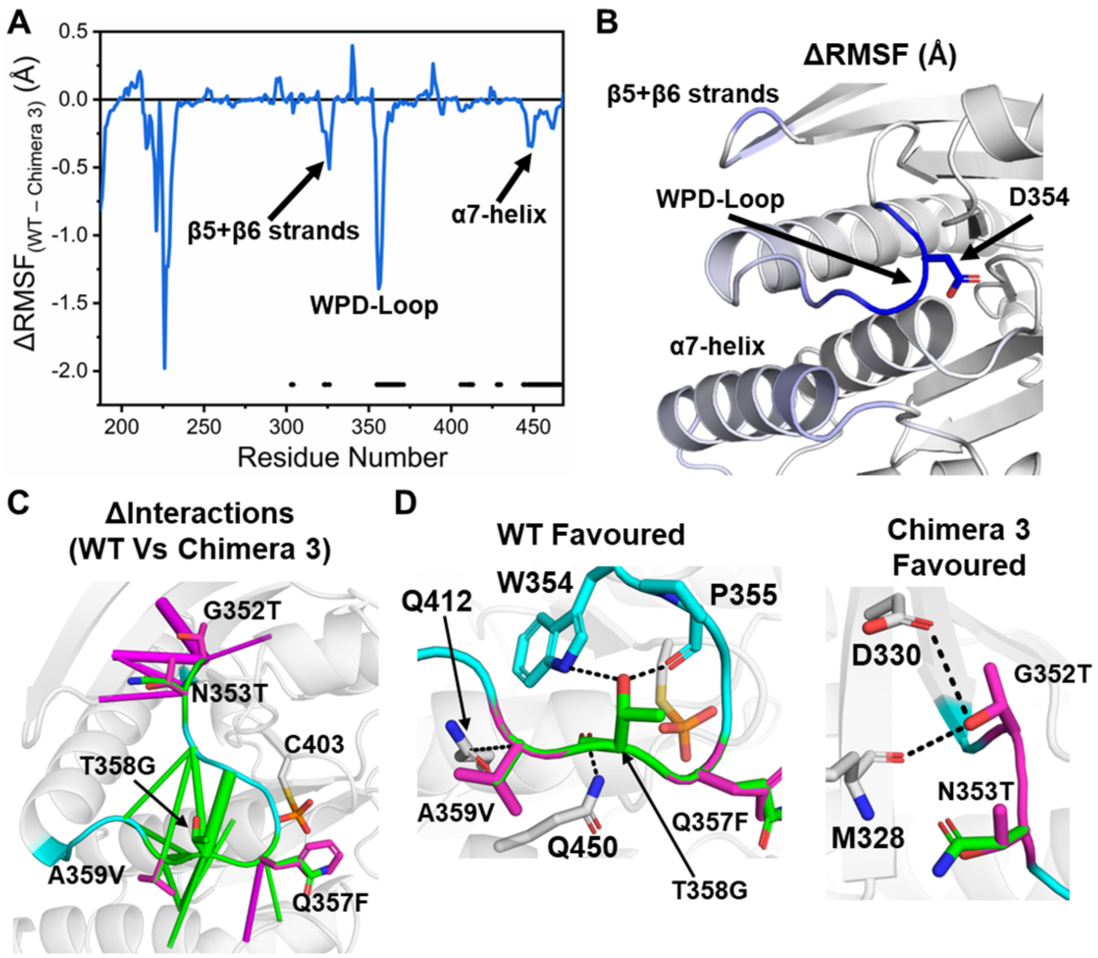

Figure S9. (**A**) Difference in the calculated root mean squared fluctuations (ΔRMSF) of wild-type YopH and Chimera 3 when sampling the closed WPD-loop conformational state. A two-sided *t*-test was performed to validate the significance of the calculated ΔRMSFs, with those residues identified as significant have a black dot placed at the bottom of the graph. To account for the usage of multiple *t*-tests, the Benjamini-Hochberg correction (Benjamini and Hochberg 1995) was applied, using a false discovery rate of 5%, see the **Supplementary Methods** for further details. (**B**) Projection of the calculated ΔRMSFs identified as significantly different onto the structure of YopH, with residues colored from blue (more rigid in the wild-type), to white (equally rigid or no significant difference) to red (more rigid in Chimera 3). (**C**) Differences in the non-covalent interaction network between wild-type YopH and Chimera 3 when sampling the closed WPD-loop

conformational state as determined by KIF (Crean et al. 2023). Interactions which are on average stronger in the wild-type are colored green, whilst those that favor Chimera 3 are colored magenta. This data is shown graphically in **Figure S11**. (**D**) Illustration of the major interactions shown in panel **C** which are on average stronger in either wild-type YopH and Chimera 3.

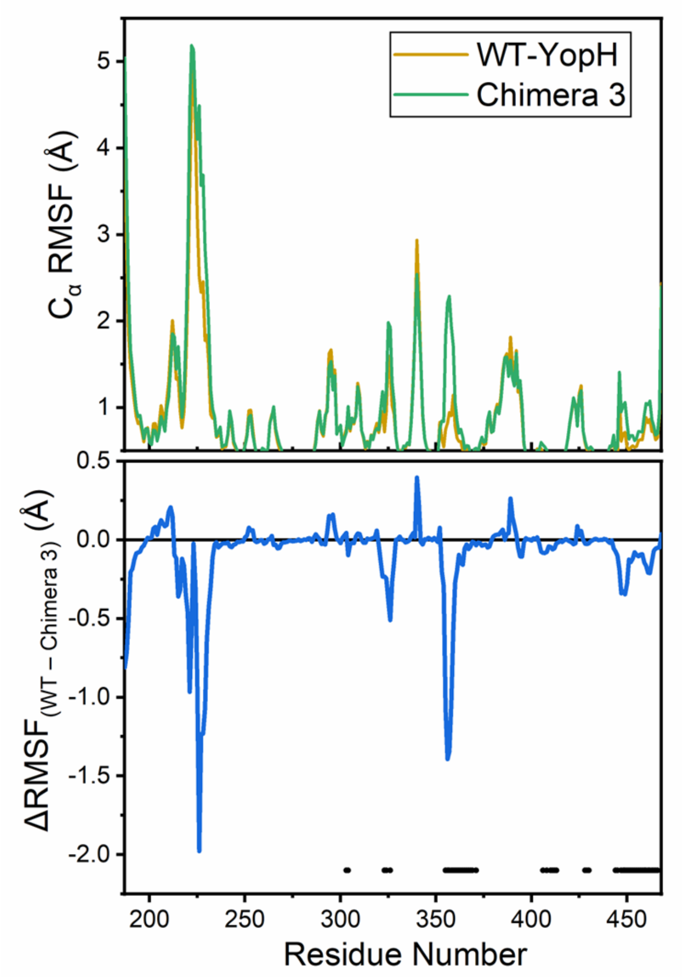

Figure S10. Comparison of the difference in the C_{α}-atom root mean squared fluctuations (RMSFs) for wild-type (WT) YopH and Chimera 3, when sampling the closed WPD-loop conformation. The top panel shows the average C_{α} RMSFs determined for each residue from 15 replicas performed per system. The bottom panel is the difference in the RMSF between both enzymes. A two-sided *t*-test was performed to determine if the differences observed for each residue was significantly different, with those identified as significant marked with a black dot. To account for the use of multiple *t*-tests, the Benjamini-Hochberg correction was applied (Benjamini and Hochberg 1995), setting the false discovery rate set to 5%, see the **Supplementary Methods** for further details.

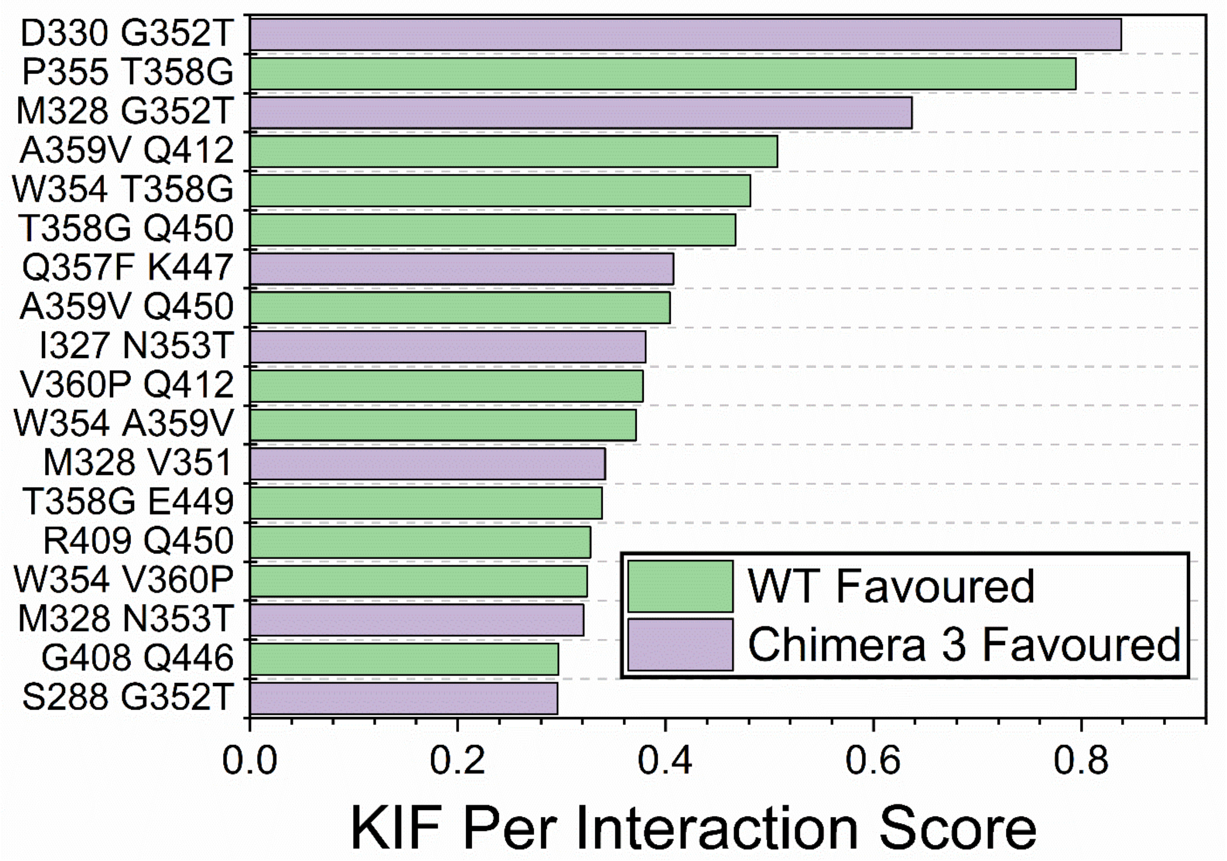

Figure S11. The largest differences in the non-covalent interaction network between wild-type (WT) YopH and Chimera 3 when sampling the closed WPD-loop conformational state, as determined by the program Key Interactions Finder (KIF) (Crean et al. 2023). All KIF scores ≥ 0.3 are shown and interactions are colored according to if they are on average stronger in WT YopH or Chimera 3.

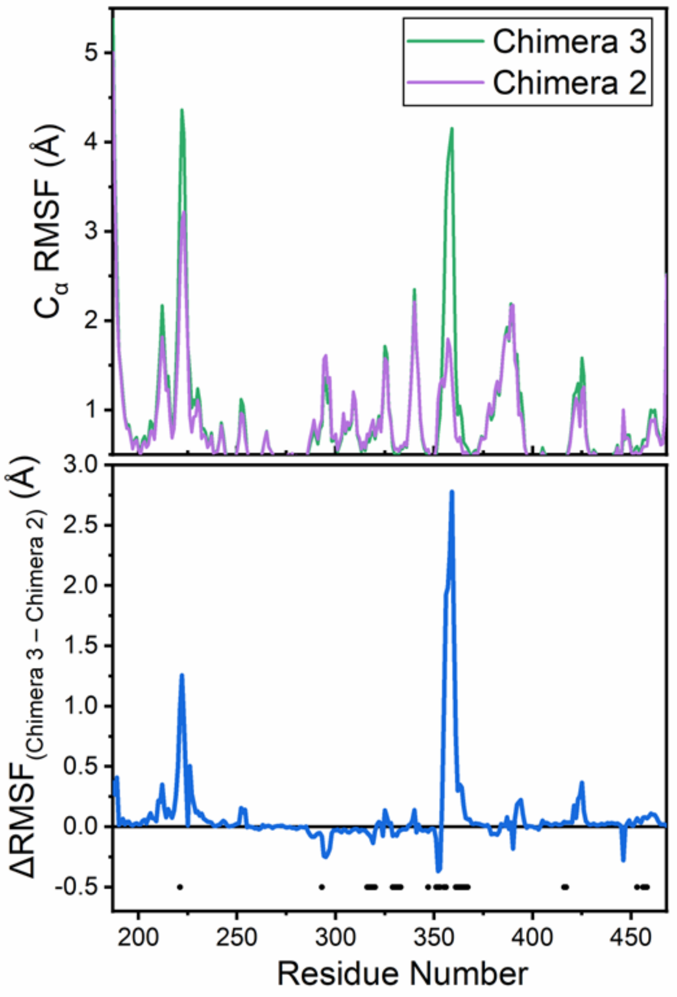

Figure S12. Comparison of the difference in the C_{α}-atom root mean squared fluctuations (RMSFs) for Chimera 3 and Chimera 2, when sampling the wide-open WPD-loop conformation. The top panel shows the average C_{α}-atom RMSFs determined for each residue from 15 replicas performed per system. The bottom panel is the difference in the RMSF between both enzymes. A two-sided *t*-test was performed to determine if the differences observed for each residue was significantly different, with those identified as significant marked with a black dot. To account for the use of multiple *t*-tests, the Benjamini-Hochberg correction was applied (Benjamini and Hochberg 1995), setting the false discovery rate set to 5%, see the **Methods section** for further details.

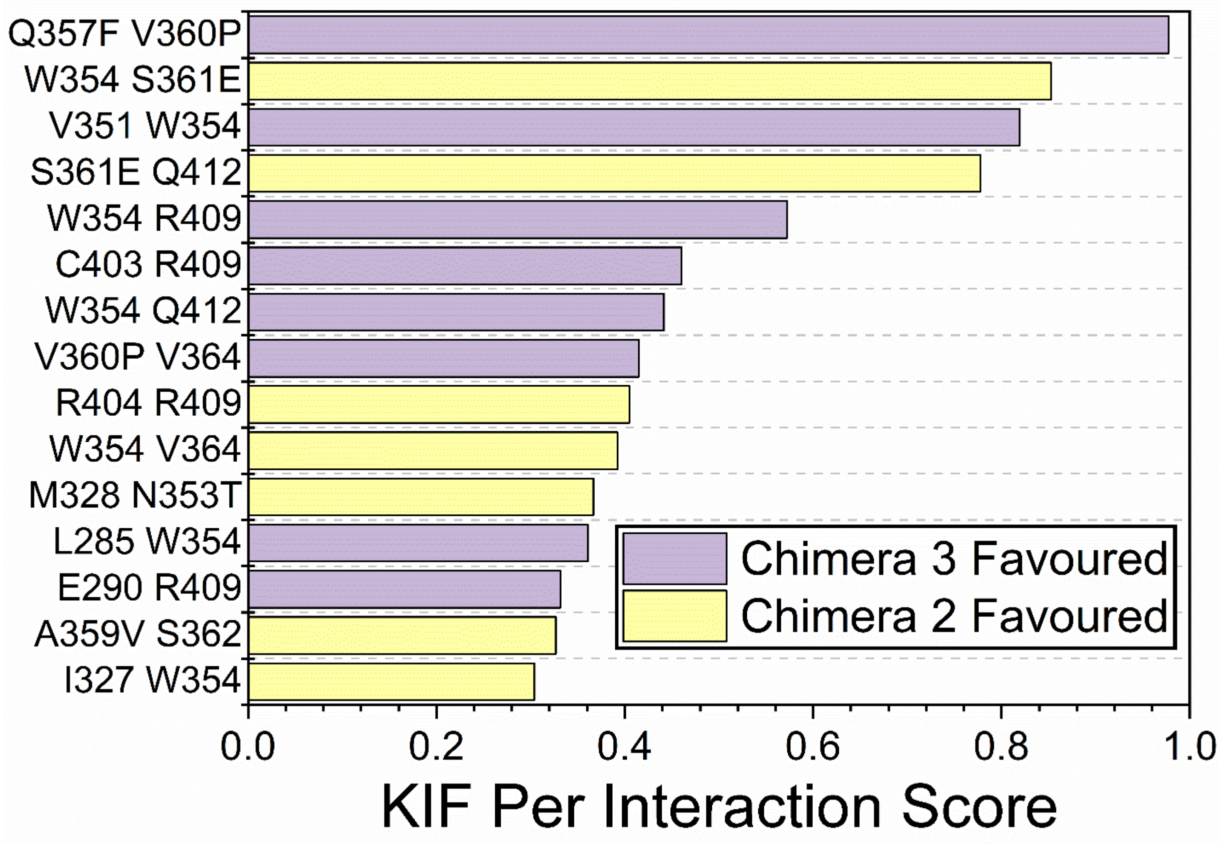

Figure S13. The largest differences in the non-covalent interaction network between Chimera 3 and Chimera 2 when sampling the wide-open WPD-loop conformational state, as determined by the program Key Interactions Finder (KIF) (Crean et al. 2023). All KIF scores ≥ 0.3 are shown and interactions are colored according to if they are on average stronger in Chimera 3 or Chimera 2.

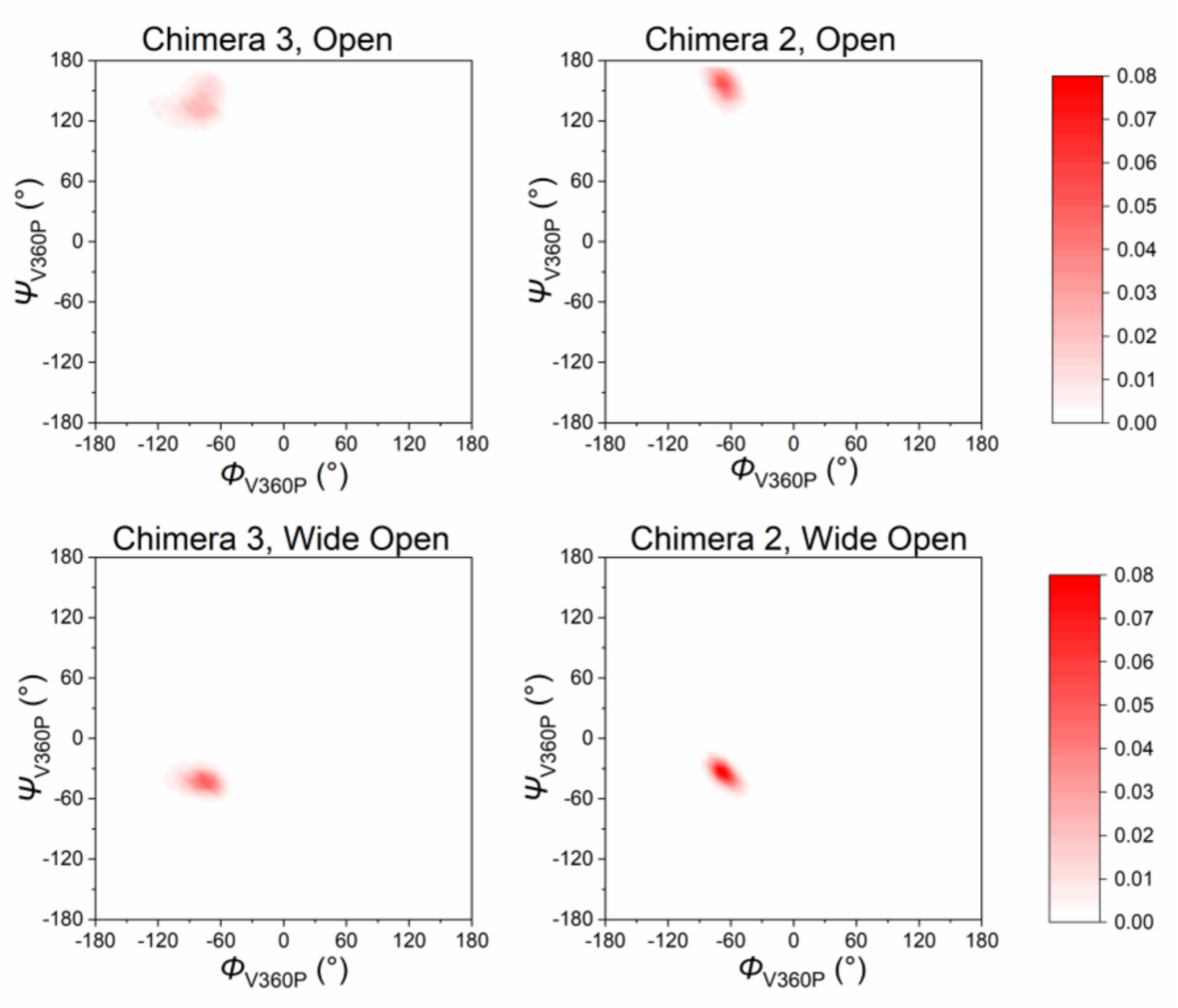

Figure S14. Normalized 2D histograms of the backbone dihedral angles adopted by V360P (valine in Chimera 3 and proline in Chimera 2). Each graph is labelled according to which enzyme is shown and the conformational state of the enzyme (wide-open or open). 2D histograms were generated using 60 bins between +180° and -180° in each dimension and normalized such that the sum of all the grid points is equal to 1.

S3. Supplementary Tables

Table S1. Calculated activation and reaction free energies from EVB simulations of wild-type PTP1B and YopH, YopH Chimeras 2 and 3, as well as the corresponding experimental parameters.[a]

	$\Delta G^{\ddagger}_{calc}$	$\Delta G_{0,calc}$	**Experimental data**			
			***k* (s^{-1})**	**Temp (°C)**	**pH**	$\Delta G^{\ddagger}_{exp}$
**Hydrolysis**						
YopH WT	14.1 ± 0.2	-2.9 ± 0.3	1234(Zhang et al. 1992)	30	5	13.5
Chimera 2	13.8 ± 0.3	-4.8 ± 0.4	0.13 (Moise et al. 2018)	23	5.5	>18.7
Chimera 3	13.7 ± 0.3	-4.6 ± 0.4	5.1(Moise et al. 2018)	23	5.5	16.4
PTP1B WT	14.3 ± 0.2	-1.4 ± 0.4	15.4(Moise et al. 2018; Stuckey et al. 1994; Whittier et al. 2013)	30	5	15.4

[a] All calculated values ($\Delta G^{\ddagger}_{calc}$ and $\Delta G_{0,calc}$) are averages and standard errors of the mean over 30 individual EVB trajectories per system, as described in the **Methods**. All energies are presented in kcal mol^{-1}. Shown here are also the corresponding experimental data, specifically the kinetics (*k*, s^{-1}), and the activation free energies ($\Delta G^{\ddagger}_{exp}$) derived from the experimentally observed rates using the Eyring equation. EVB data for YopH and PTP1B are from our prior work (Crean et al. 2022; Crean et al. 2021). WT denotes the wild-type parent enzymes.

Table S2. Calculated distances at the Intermediate states (IS), transition states (TS) and product states (PS) obtained from EVB simulations. Shown are also the average number of water molecules within 4Å of the reacting atoms (P and S_{Cys}).[a]

System		S_{Cys}-P	P-O_{H2O}	O_{H2O}-H	H-O_{Asp}	No. of waters
**YopH WT**	**IS**	1.97 ± 0.01	3.18 ± 0.03	0.99 ± 0.01	2.33 ± 0.19	1.9 ± 0.0
	**TS**	2.33 ± 0.02	2.12 ± 0.02	1.08 ± 0.01	1.24 ± 0.01	2.7 ± 0.1
	**PS**	3.28 ± 0.03	1.62 ± 0.01	1.34 ± 0.01	1.01 ± 0.01	3.1 ± 0.1
**Chimera 2**	**IS**	1.97 ± 0.01	3.17 ± 0.11	0.97 ± 0.01	2.49 ± 0.20	2.2 ± 0.1
	**TS**	2.35 ± 0.02	2.12 ± 0.02	1.08 ± 0.01	1.25 ± 0.01	2.7 ± 0.1
	**PS**	3.33 ± 0.03	1.62 ± 0.01	1.34 ± 0.01	1.00 ± 0.01	3.1 ± 0.2
**Chimera 3**	**IS**	1.97 ± 0.01	3.41 ± 0.18	0.97 ± 0.01	2.42 ± 0.19	2.4 ± 0.1
	**TS**	2.35 ± 0.02	2.11 ± 0.02	1.08 ± 0.01	1.24 ± 0.01	2.9 ± 0.1
	**PS**	3.34 ± 0.03	1.62 ± 0.01	1.34 ± 0.01	1.01 ± 0.01	3.1 ± 0.2
**PTP1B WT**	**IS**	1.97 ± 0.01	3.17 ± 0.06	0.99 ± 0.01	2.06 ± 0.19	1.6 ± 0.1
	**TS**	2.32 ± 0.02	2.11 ± 0.02	1.08 ± 0.01	1.25 ± 0.01	2.2 ± 0.1
	**PS**	3.32 ± 0.03	1.61 ± 0.01	1.34 ± 0.02	1.01 ± 0.01	2.5 ± 0.1

[a] IS, TS and PS correspond to the phospho-enzyme intermediate, transition state and product state for the hydrolysis step, respectively. $S_{Cys\text{-}P}$ denotes the distance between the cysteine side-chain and the phosphorus atom of the phosphate group, P-O_{H2O} denotes the distance between the phosphorus atom and the nucleophilic water molecule in the hydrolysis step, O_{H2O}-H denotes the distance between the nucleophilic water molecule and the proton being transferred back to the aspartic acid side-chain, and H-O_{Asp} denotes the distance between the proton and the relevant oxygen atom of the aspartic acid side-chain. All distances are shown in Å. Data is presented as average values and standard error of the mean over 30 individual EVB trajectories per system. "WT" denotes the wild-type enzyme. YopH and PTP1B wild-type data was originally presented in refs. (Crean et al. 2022; Crean et al. 2021).

Table S3. Electrostatic contributions of selected amino acids ($\Delta\Delta G^{\ddagger}_{elec}$, kcal mol^{-1}) to the calculated activation free energies for the hydrolysis steps catalyzed by wild-type (WT) YopH and Chimeras 2 and 3.[a]

Residue	WT-YopH	Chimera 2	Chimera 3
E290	-1.18 ± 0.03	-0.88 ± 0.02	-0.84 ± 0.01
Q/F357	0.89 ± 0.05	0.14 ± 0.01	0.05 ± 0.02
R398	-0.21 ± 0.00	-0.22 ± 0.00	-0.21 ± 0.00
R404	1.53 ± 0.13	1.51 ± 0.11	1.41 ± 0.12
G406	-0.50 ± 0.01	-0.53 ± 0.00	-0.49 ± 0.00
V407	-0.16 ± 0.01	-0.17 ± 0.00	-0.15 ± 0.00
G408	-0.15 ± 0.01	-0.16 ± 0.00	-0.15 ± 0.00
R409	-0.65 ± 0.03	-0.61 ± 0.02	-0.56 ± 0.01
T410	-0.82 ± 0.05	-0.82 ± 0.01	-0.77 ± 0.00
R440	-0.62 ± 0.01	-0.68 ± 0.00	-0.63 ± 0.00
K447	0.51 ±0.03	0.51 ± 0.01	0.52 ± 0.03
E449	-0.24 ± 0.01	-0.26 ± 0.01	-0.24 ± 0.01

[a] All electrostatic contributions were scaled assuming an internal dielectric constant of 4. (Li et al. 2013) Data was obtained from the calculated EVB trajectories using the linear response approximation (LRA) and is represented as average and standard error of the mean over 30 individual trajectories per system (Lee et al. 1992; Muegge et al. 1997). Note that only residues that have $\Delta\Delta G^{\ddagger}_{elec} \geq 0.30$ kcal mol^{-1} are shown here individually for clarity.

Table S4. List of ionized residues and histidine protonation patterns used in our EVB simulations of YopH chimeras 2 and 3.[a]

Residue Type	Residue Number
Asp	231, 300, 330, 356, 448, 452
Glu	290, 361[b], 449
Arg	194, 05, 228, 230, 295, 303, 398, 404, 409, 437, 440
Lys	225, 447
His-ε	402
His-δ	270, 350

[a] The ionizable residues that fell outside of the explicit simulation sphere were kept in their neutral forms to avoid system instabilities created by having changed residues outside the water droplet (this is standard practice for such simulations). All other residues were kept in their usual ionization state at physiological pH. In the case of the histidine side-chains, His-ε and His-δ indicate histidine side-chains protonated at the $N_{\varepsilon 2}$ and $N_{\delta 1}$ nitrogen atoms, respectively.

[b] Glu361 is only present in Chimera 2.

Table S5. Starting structures used for the molecular dynamics (MD) and targeted MD (tMD) simulations of WT YopH.[a]

Simulation	PDB ID	Modifications required before simulation.
Closed	2I42 (Denu et al. 1996)	Bound vanadate ion modified into a phosphate group, geometry optimized.
Open	1YPT (Stuckey et al. 1994)	Phospho group attached to catalytic cysteine residue.
Wide-open	Two MD simulation generated structures of WT YopH generated from prior simulations. [a]	Phospho group attached to catalytic cysteine residue.

[a] With no crystal structures available of WT YopH in the wide-open state, we used structures we generated from our prior simulations (Crean et al. 2021) of WT-YopH using an enhanced sampling MD simulation approach. See the **Methods** section for further details.

Table S6. Starting structures used for the molecular dynamics (MD) and targeted MD (tMD) simulations of Chimera 3.

**Simulation**	**PDB ID**	**Modifications required before simulation.**
Closed	2I42 (Denu et al. 1996)	Steps described for WT YopH and WPD-loop substitutions made to convert from WT to Chimera 3 sequence.
Open	6DR9 (Moise et al. 2018)	Phospho group attached to catalytic cysteine residue
Wide-Open	6DT6 (Moise et al. 2018)	Vanadate ion in the active site exchanged to a phospho group that is attached to the catalytic cysteine residue

Table S7. Starting structures used for the Molecular dynamics (MD) and Targeted MD (tMD) simulations of Chimera 2.

**Simulation**	**PDB ID**	**Modifications required before simulation.**
Closed	2I42 (Denu et al. 1996)	Optimisation described for WT YopH and WPD-loop substitutions made to convert from WT to Chimera 2 sequence.
Open	6DR9 (Moise et al. 2018)	Steps taken for Chimera 3 open state alongside substitutions needed to convert from Chimera 3 to Chimera 2
Wide-open	6DR7 (Moise et al. 2018)	Vanadate ion in the active site exchanged to a phospho group that is attached to the catalytic cysteine residue

S4. Supplementary References

Benjamini Y and Hochberg Y (1995) Controlling the False Discovery Rate: A Practical and Powerful Approach to Multiple Testing. *J. R. Stat. Soc. Series B Stat. Methodol.* 57, 289-300.

Berendsen HJC, Postma JPM, van Gunsteren WF, DiNola A and Haak JR (1984) Molecular Dynamics with Coupling to an External Bath. *J. Chem. Phys.* 81, 3684-3690.

Berman HM, Westbrook J, Feng Z, Gilliland G, Bhat TN, Weissig H, Shindyalov IN and Bourne PE (2000) The Protein Data Bank. *Nucleic Acids Res.* 28, 235-242.

Bussi G (2014) Hamiltonian Replica Exchange in GROMACS: A Flexible Implementation. *Mol. Phys.* 112, 379-384.

Crean RM, Biler M, Corbella M, Calixto AR, van der Kamp M, Hengge AC and Kamerlin SCL (2022) Correction to "Loop Dynamics and Enzyme Catalysis in Protein Tyrosine Phosphatases". *J. Am. Chem. Soc.* 144, 10091-10093.

Crean RM, Biler M, van der Kamp MW, Hengge AC and Kamerlin SCL (2021) Loop Dynamics and Enzyme Catalysis in Protein Tyrosine Phosphatases. *J. Am. Chem. Soc.* 143, 3830-3845.

Crean RM, Slusky JSG, Kasson PM and Kamerlin SCL (2023) KIF – Key Interactions Finder: A Program to Identify the Key Molecular Interactions that Regulate Protein Conformational Changes. *J. Chem. Phys.* 158, 144114.

Denu JM, Lohse DL, Vijayalakshmi J, Saper MA and Dixon JE (1996) Visualization of Intermediate and Transition-State Structures in Protein-Tyrosine Phosphatase Catalysis. *Proc. Natl. Acad. Sci. USA* 93, 2493-2498.

Lee FS, Chu Z-T, Bolger MB and Warshel A (1992) Calculations of Antibody-Antigen Interactions: Microscopic and Semi-Microscopic Evaluation of the Free Energies of Binding of Phopshorylcholine Analogs to McPC603. *Protein Eng. Des. Sel.* 5, 215-228.

Li L, Li C, Zhang Z and Alexov E (2013) On the Dielectric "Constant" of Proteins: Smooth Dielectric Function for Macromolecular Modeling and Its Implementation in DelPhi. *J. Chem. Theory Comput.* 9, 2126-2136.

Moise G, Morales Y, Beaumont V, Caradonna T, Loria JP, Johnson SJ and Hengge AC (2018) A YopH PTP1B Chimera Shows the Importance of the WPD-Loop Sequence to the Activity, Structure and Dynamics of Protein Tyrosine Phosphatases. *Biochemistry* 57, 5315-5326.

Muegge I, Tao H and Warshel A (1997) A Fast Estimate of Electrostatic Group Contributions to the Free Energy of Protein-Inhibitor Binding. *Protein Eng. Des. Sel.* 10, 1363-1372.

Pettersen EF, Goddard TD, Huang CC, Gouch CS, Greenblatt DM, Meng EC and Ferrin TE (2004) UCSF Chimera - A Visualization System for Exploratory Research and Analysis. *J. Comput. Chem.* 25, 1605-1612.

Roe DR and Cheatham TE (2013) PTRAJ and CPPTRAJ: Software for Processing and Analysis of Molecular Dynamics Trajectory Data. *J. Chem. Theory. Comput.* 9, 3084-3095.

Ryckaert J-P, Ciccotti G and Berendsen HJC (1977) Numerical Integration of the Cartesian Equations of Motion of a System with Constraints: Molecular Dynamics of *n*-Alkanes. *J. Comput. Phys.* 23, 327-341.

Scheurer M, Rodenkirch P, Siggel M, Bernardi RC, Schulten K, Tajkhorshid E and Rudack T (2018) PyContact: Rapid, Customizable, and Visual Analysis of Noncovalent Interactions in MD Simulations. *Biophys. J.* 114, 577-583.

Schneider T and Stoll E (1978) Molecular-Dynamics Study of a Three-Dimensional One-Component Model for Distortive Phase Transitions. *Phys. Rev. B* 17, 1302-1322.

Shapovalov MX and Dunbrack Jr. RL (2011) A Smoothed Backbone-Dependent Rotamer Library for Proteins Derived from Adaptive Kernel Density Estimates and Regressions. *Structure* 19, 844-858.

Shen R, Crean RM, Olsen KJ, Corbella M, Calixto AR, Richan T, Brandão TAS, Berry RD, Tolman A, Loria JP, Johnson SJ, Kamerlin SCL and Hengge AC (2022) Insights into the Importance of WPD-Loop Sequence in Protein Tyrosine Phosphatases. *Chem. Sci.* 13, 13524-13540.

Stuckey JA, Schubert HL, Fuaman EB, Zhang Z-Y, Dixon JE and Saper MA (1994) Crystal Structure of Yersinia Protein Tyrosine Phosphatase at 2.5 Å and the Complex with Tungstate. *Nature* 370, 571-575.

Warshel A and King G (1985) Polarization Constraints in Molecular Dynamics Simulation of Aqueous Solutions: The Surface Constraint All Atom Solvent (SCAAS) Model. *Chem. Phys. Lett.* 121, 124-129.

Warshel A and Weiss RM (1980) An Empirical Valence Bond Approach for Comparing Reactions in Solutions and in Enzymes. *J. Am. Chem. Soc.* 102, 6218-6226.

Whittier SK, Hengge AC and Loria JP (2013) Conformational Motions Regulate Phosphoryl Transfer in Related Protein Tyrosine Phosphatases. *Science* 341, 899-903.

Zhang ZY, Clemens JC, Schubert HL, Stuckey JA, Fischer MW, Hume DM, Saper MA and Dixon JE (1992) Expression, Purification, and Physicochemical Characterization of a Recombinant Yersinia Protein Tyrosine Phosphatase. *J. Biol. Chem.* 267, 23759-23766.
